# Satisfaction of adult and pediatric neurologists and neurosurgeons using telehealth during the COVID-19 pandemic in Saudi Arabia: a cross-sectional study

**DOI:** 10.3389/fdgth.2024.1195697

**Published:** 2024-02-14

**Authors:** Saleh Ayed Algarni, Maha Hamoud Alrashid, Mohammed Sultan Aldayel, Lujain Habeeb Allowaihiq, Abdulaziz Ali Almuqbil, Anas Mohammad Albarrak, Sulaiman Almobarak

**Affiliations:** ^1^ Department of Neuroscience, King Faisal Specialist Hospital & Research Center, Riyadh, Saudi Arabia; ^2^ College of Medicine, Alfaisal University, Riyadh, Saudi Arabia; ^3^ College of Medicine, Imam Mohammad Ibn Saud Islamic University, Riyadh, Saudi Arabia; ^4^ Department of Internal Medicine, College of Medicine, Prince Sattam Bin Abdulaziz University, Al-Kharj, Saudi Arabia; ^5^ Pediatric Neurology Department, King Saud Medical City, Riyadh, Saudi Arabia

**Keywords:** telehealth, satisfaction, COVID-19, neurologist, neurosurgeon telehealth, pediatric neurologist, neurosurgeon

## Abstract

**Objectives:**

Telehealth has become increasingly important in achieving universal health coverage. It offers doctors and their patients' convenience, including providing quality care at reduced costs. During the coronavirus disease (COVID)-19 pandemic, telehealth has been a vital tool for remote healthcare services. This study aimed to assess the satisfaction of adult and pediatric neurologists and neurosurgeons using telehealth, during the COVID-19 pandemic in Saudi Arabia.

**Methods:**

This study had 348 participants. It was conducted among adult and pediatric neurologists and neurosurgeons using telehealth technology at their clinics between February and June 2021. The self-administered questionnaire included sociodemographic data, behavior in using telehealth, and an assessment of satisfaction with telehealth; the SPSS Windows software version 26 was used to analyze the data.

**Results:**

The most common age group was 25–34 years (42.8%), with men dominating (68.4%). The mean satisfaction score was 25.9 (SD 3.91) out of 33 points, with 90.2% of respondents satisfied with telehealth and 9.8% dissatisfied. Working in an academic center or private hospital, being a first-time telehealth user, using messages as a telehealth method, and using telehealth daily were associated with increased satisfaction with telehealth use.

**Conclusion:**

The satisfaction of adult and pediatric neurologists and neurosurgeons with telehealth was high. Although physicians still preferred face-to-face interviews, they recognized the benefits of telehealth in strengthening the patient–provider relationship, improving productivity, and integrating into daily workflows. The satisfaction levels align with past studies, but physical examination needs should be considered. Telehealth is suitable for follow-up visits and varies across subspecialties.

## Introduction

Telehealth is defined as the remote provision of healthcare services to patients (1). It utilizes technology for diagnosis, treatment, research, and education in healthcare. It aims to provide high-quality and affordable services globally, benefiting those in remote areas, vulnerable populations, and the elderly. Its aim is to achieve universal health coverage (1). Telemedicine is utilized in various medical scenarios, from emergency situations to long-term management of chronic neurological conditions. It enables remote transmission of clinical information like vital signs and audio/video, from patients' homes to healthcare providers via the internet or telephone lines (2, 3). Telemedicine encompasses various channels, including the telephone, video conferencing, and smartphones, making healthcare more accessible. It comprises synchronous interactions between patients and providers, as well as asynchronous methods like email, remote monitoring, and store-and-forward techniques. In the store-and-forward approach, clinical information is collected and shared with a doctor for evaluation and consultation, including images, electroencephalography (EEG) traces, or case histories (2). TeleStroke, an early application of teleneurology, has become a standard practice for acute neurological care. Prior to TeleStroke, many patients with stroke symptoms went without evaluation by a neurologist, and less than 1.5% of acute stroke patients received thrombolytic treatment (4). The stroke initiative, led by the American Heart Association/American Stroke Association, has successfully improved patient access to emergency neurological treatment. As a result, a significant number of eligible patients are now receiving IV tPA, a clot-dissolving medication (5). TeleStroke has advanced beyond mobile computed tomography (CT)-enabled ambulances to provide hospital-level care directly to patients (6). Furthermore, TeleStroke appears to be safe and cost-effective when compared with usual care (7, 8). With improved education, the capacity of local hospitals worldwide to manage acute stroke cases has been enhanced (2).

Before the coronavirus disease (COVID)-19 pandemic, telemedicine adoption has been gradually increasing, primarily for primary and mental healthcare. However, the pandemic necessitated social distancing and clinic closures, prompting a shift toward telemedicine to meet patients' needs for appointments and follow-ups. Governments implemented supportive laws and regulations to facilitate telemedicine and slow the spread of the disease (9).

Several studies have examined patient and provider satisfaction in telehealth. In a study with 168 patients, telemedicine was associated not only with patient satisfaction but also with increased investigations (10). In a study of pediatric neurologists during the COVID-19 pandemic, telehealth was found to have been used for 2,589 children, while 14,780 in-person visits were made. Provider satisfaction with telemedicine was high in 93% of the interactions. Furthermore, it is preferred as a mode of follow-up (11). Another study during the pandemic involving neurosurgeons and their patients in the USA found that 92% of patients strongly agreed or agreed that they were satisfied with virtual consultations. In addition, 88% of patients stated that virtual consultations were more convenient for them (12). Among local studies in Saudi Arabia, a cross-sectional study titled “Implementation of Virtual Consultations for Epilepsy during the COVID-19 Pandemic among Neurologists in Saudi Arabia” concluded that there was a statistical significance in the interaction time between virtual consultation and on-site consultation (13). Another study that was conducted to identify the advantages and disadvantages of using telehealth during the COVID-19 pandemic states that one of the obstacles to reaching a diagnosis is the lack of physical examination (14). Yet another study of 425 patients reported that almost half of the patients were satisfied. They found a positive attitude toward telemedicine programs in Saudi Arabia (15). Since various studies in the literature illustrate patient satisfaction, it is also crucial to understand the perspectives of physicians on telemedicine. However, few studies have specifically addressed the satisfaction of adult and pediatric neurologists and neurosurgeons with telemedicine. The existing studies tend to focus more on their opinions regarding the advantages and disadvantages of telehealth rather than their overall satisfaction.

Therefore, we conducted a study in Saudi Arabia to assess the satisfaction of neurologists and neurosurgeons with telehealth during the COVID-19 pandemic. Our aim was to evaluate the ease of using telehealth, the quality of communication between physicians and patients, and understand how the services were perceived. The study included adult and pediatric practitioners in the medical and surgical fields, to better represent the overall experience. Telehealth has the potential to provide timely, cost-effective, and high-quality care, especially in situations where geographic, economic, and physical limitations, as well as social distancing measures, hinder access to specialty care.

## Methodology

### Study participants

This study used a descriptive cross-sectional questionnaire-based design. It was conducted in Saudi Arabia from February to June 2021. A total of 359 physicians were randomly selected and asked to participate in an online survey. The inclusion criteria were an adult or pediatric neurologist or neurosurgeon above 25 years of age who used telehealth technology, in adult or pediatric neurology or neurosurgery clinics, during the COVID-19 pandemic in Saudi Arabia. All participants received an online web page explaining the study purpose and were requested to provide informed consent before completing the online questionnaire.

### Sample size

According to the Saudi Commission for Health Specialties, 1,761 adult and pediatric neurologists and neurosurgeons lived in Saudi Arabia in 2020. Based on an acceptable error margin of 5% with a confidence interval of 95%, a sample size of at least 316 was required. The dataset included 359 participants. We excluded participants who refused to participate and those who had never used telehealth. The final sample size after exclusion was 348.

### Data collection tool

The questionnaire was adapted with permission from Becevic et al*.* (16). Response repetition was prevented by linking every survey response with an Internet protocol. Further, the Medical College Institutional Review Board of Al-Imam Muhammad Ibn Saud Islamic University, Riyadh, Saudi Arabia, approved the study protocol – approval number 37-2021.

### Statistical analysis

The data were collected from an online platform and analyzed using the Statistical Packages for Social Sciences (SPSS) Windows software version 26. The satisfaction with using telehealth during the COVID-19 pandemic was measured using an 11-item questionnaire with “disagree” coded with 1, “neutral” coded with 2, and “agree” coded with 3 being the answer options. Item 10 was classified as a negative question, and the answer was revised accordingly to avoid bias in calculating the score. The total satisfaction score was calculated by adding all 11 items. A possible score range of 11–33 points was generated, indicating that the higher the score, the higher the satisfaction with using telehealth. Based on the Standard Setting Simplified—Cohen, using 60% of the total score points to determine the level of satisfaction, adult or pediatric neurologists or neurosurgeons were considered dissatisfied if the score was 60% or below of the total score, and satisfied if the score was above 60% (17). The mean, standard deviation, and median (interquartile range) presented quantitative data, whereas numbers and percentages were used to summarize the qualitative data. The satisfaction score was compared with the sociodemographic characteristics and the behavior toward telehealth using the Mann–Whitney *U*-test and Kruskal–Wallis test, as applied. The data's normality and homogeneity were tested using the Shapiro–Wilk test. The satisfaction scores followed a non-normal distribution, and non-parametric tests were applied. A *p*-value cutoff point of 0.05 at 95% CI was used to determine statistical significance.

## Results

This study recruited 348 adult and pediatric neurologists and neurosurgeons. Table 1 presents the sociodemographic characteristics of the participants. The most common age group was 25–34 years (42.8%), with males dominating (68.4%), and the majority being Saudis (90.8%). Concerning the type of hospital, nearly 70% worked at government hospitals, primarily because these hospitals serve as the main healthcare providers in Saudi Arabia. Furthermore, almost half (50.9%) of the participants resided in the central region, which encompass the highest overall number of physicians all over the Kingdome of Saudi Arabia. Regarding marital status, approximately three-quarters of the participants were married (73%). The consultants constituted 56% of the total, whereas the residents constituted 30.2%. Approximately 60% were adult neurologists, 20.7% were pediatric neurologists, and 19.5% were neurosurgeons.

**Table 1:** Sociodemographic characteristics of adult and pediatric neurologists and neurosurgeons (*n* = 348).

Study data	*n* (%)
Age group	
• 25–34 years	149 (42.8)
• 35–44 years	96 (27.6)
• 45–54 years	85 (24.4)
• 55–64 years	18 (5.2)
Gender	
• Men	238 (68.4)
• Women	110 (31.6)
Nationality	
• Saudi	316 (90.8)
• Non-Saudi	32 (9.2)
Type of hospital	
• Private hospital	63 (18.1)
• Government hospital	241 (69.3)
• Academic center	41 (11.8)
• Academic and private hospital	2 (0.6)
• Currently unemployed	1 (0.3)
Residence region	
• Central region	177 (50.9)
• Western region	112 (32.2)
• Eastern region	36 (10.3)
• Southern region	16 (4.6)
• Northern region	07 (2.0)
Marital status	
• Never been married	78 (22.4)
• Married	254 (73.0)
• Engaged	5 (1.4)
• Divorced	11 (3.2)
Position	
• Resident	105 (30.2)
• Registrar or senior registrar	25 (7.2)
• Fellow	23 (6.6)
• Consultant	195 (56.0)
Specialty	
• Neurology	208 (59.8)
• Neurosurgery	68 (19.5)
• Pediatric neurology	72 (20.7)

Table 2 shows the behavior of physicians using telehealth services. It can be observed that nearly 70% were first-time users of telehealth. The most commonly used method for telehealth was voice calls (89.9%), followed by messages (67.2%) and video calls (33.9%). In addition, 54.6% used telehealth weekly, and 38.8% used it every day.

**Table 2:** Telehealth use by adult and pediatric neurologists and neurosurgeons (*n* = 348).

Variables	*n* (%)
I have been using telehealth for	
• First time during the COVID-19 pandemic	243 (69.8)
• 1–2 years	78 (22.4)
• 3–5 years	16 (4.6)
• >5 years	11 (3.2)
Method used for telehealth^a^	
• Voice call	313 (89.9)
• Video call	118 (33.9)
• Messages	234 (67.2)
I use/used telehealth	
• Every day	135 (38.8)
• Weekly	190 (54.6)
• Monthly	13 (3.7)
• Only during lockdown	2 (0.60)
• Once a year	8 (2.3)

^a^ Variable with multiple response answers.

The assessment of satisfaction with telehealth use during the COVID-19 pandemic among physicians is given in Table 3. Following the results, we observed that 29% of the respondents agreed that they did not face difficulties using the telehealth system, while 22.1% did. A total of 44.3% of participants agreed that they felt comfortable using the telehealth system, but 08.3% did not. Furthermore, 54.3% agreed that telehealth could provide opportunities to develop good relationships with their patients. Nearly half agreed that telehealth fits well with the daily work process, facilitates seeing more patients, allows for more time to talk to patients, and is satisfactory in terms of audio and video quality during interactions with patients. Only 36.2% of participants claimed to effectively address the needs of their patients, leaving a significant gap in healthcare provision. Astonishingly, more than half of the participants expressed their disagreement with the notion that telehealth visits surpass the efficacy of traditional face-to-face encounters. Simultaneously, 60.1% agreed that they are generally happy with the work they have accomplished using telehealth. Based on the 11-item questionnaire, the mean satisfaction score was 25.9 (SD 3.91) out of 33 points (median: 27; IQR: 5.00).

**Table 3:** Assessment of adult and pediatric neurologists’ and neurosurgeons’ satisfaction with using telehealth during the COVID-19 pandemic (*n* = 348).

Statement	Disagree *n* (%)	Neutral *n* (%)	Agree *n* (%)
1. It is easy to run and use the telehealth system	77 (22.1)	170 (48.9)	101 (29.0)
2. I am confident and at ease when I use the telehealth system	29 (08.3)	165 (47.4)	154 (44.3)
3. Telehealth gives me the chance to build and keep a personal bond with each of my patients	23 (06.6)	136 (39.1)	189 (54.3)
4. Telehealth fits well with each day's workflow	33 (09.5)	141 (40.5)	174 (50.0)
5. The images and sounds of telehealth gear are clear and crisp	35 (10.1)	134 (38.5)	179 (51.4)
6. I accomplish more in my day when I see patients through telehealth	42 (12.1)	130 (37.4)	176 (50.6)
7. Telehealth helps me to converse with my patients	47 (13.5)	129 (37.1)	172 (49.4)
8. Telehealth allows me to see more patients	40 (11.5)	138 (39.7)	170 (48.9)
9. I am able to treat my patients’ needs well through telehealth	71 (20.4)	151 (43.4)	126 (36.2)
10. I prefer telehealth visits over visits that are in person^a^	199 (57.2)	117 (33.6)	32 (09.2)
11. For the most part, I am satisfied with the work I have carried out through telehealth	33 (9.5)	106 (30.5)	209 (60.1)
Total satisfaction score			
• Mean ± SD	• 25.9 ± 3.91		
• Median (IQR)	• 27.0 (5.00)		

^a^ Questions with reverse answers.

When measuring the difference in the perception score regarding sociodemographic characteristics and their behavior toward telehealth, statistically significant median satisfaction scores were seen in those working in academic centers/private hospitals (*U* = 6,377; *p* = 0.050), first-time users of telehealth (*U* = 11,061; *p* = 0.034), using messages for telehealth (*U* = 10.624; *p* = 0.002), and using telehealth daily (*H* = 17.174; *p* < 0.001). Contrastingly, the mean satisfaction scores for age group (*p* = 0.246), gender (*p* = 0.184), nationality (*p* = 0.303), residence region (*p* = 0.390), marital status (*p* = 0.407), position (*p* = 0.153), and specialty (*p* = 0.443) were not significantly different across the groups (Table 4).

**Table 4:** Differences in satisfaction scores in the sociodemographic characteristics and behavior toward telehealth among adult and pediatric neurologists and neurosurgeons (*n* = 348).

Factor	Satisfaction total score (33) mean (IQR)	*U*/*H* test	*p*-Value
Age group*			
• <35 years	26.0 (4.00)	*U* = 13,753.5	0.246
• ≥35 years	27.0 (5.00)		
Gender*			
• Men	27.0 (5.00)	*U* = 11,935	0.184
• Women	26.0 (4.00)		
Nationality*			
• Saudi	27.0 (5.00)	*U* = 4,500	0.303
• Non-Saudi	26.5 (3.50)		
Type of hospital^a^*			
• Academic center/private hospital	27.0 (4.00)	*U* = 6,377	**0.050** ***
• Government hospital	26.0 (4.00)		
Residence region*			
• Central region	27.0 (5.00)	*U* = 14,330.5	0.390
• Non-central region	26.0 (4.00)		
Marital status*			
• Unmarried	26.0 (4.00)	*U* = 11,249.5	0.407
• Married	27.0 (5.00)		
Position**			
• Resident	27.0 (5.00)	*H* = 3.754	0.153
• Registrar/fellow	25.5 (4.75)		
• Consultant	27.0 (5.00)		
Specialty**			
• Neurology	26.5 (5.00)	*H* = 1.627	0.443
• Neurosurgery	26.5 (4.75)		
• Pediatric neurology	27.0 (4.00)		
I have used/been using telehealth for*			
• First time during the COVID-19 pandemic	27.0 (5.00)	*U* = 11,061	**0.034** ***
• One to more than 5 years	26.0 (3.00)		
Method used for telehealth^b^*			
• Voice call	27.0 (5.00)	*U* = 5,369	0.847
• Video call	27.0 (4.00)	*U* = 12,656	0.302
• Messages	27.0 (4.00)	*U* = 10,624	**0.002** ***
I used telehealth*			
• Every day	27.0 (4.00)	*H* = 17.174	**<0.001** ***
• Weekly	26.0 (5.00)		
• Monthly or occasionally	24.0 (5.00)		

*U/H*, Mann–Whitney *U* test/Kruskal–Wallis *H* test.

^a^ Currently not working was excluded from the analysis.

^b^ Variable with multiple response options.

* *p*-Value has been calculated using the Mann–Whitney *U* test.

** *p*-Value has been calculated using the Kruskal–Wallis test.

*** Significant at *p* ≤ 0.05 level.

The bold values means significant finding.

## Discussion

The present study evaluated the satisfaction of adult and pediatric neurologists and neurosurgeons, with telehealth during the COVID-19 pandemic. In neurology, TeleStroke was among first to utilize telehealth in neurology especially for acute stroke to decrease door-to-needle time needed for administration of thrombolysis (18). This resulted in guidelines issued by both the European Stroke Organization and the American Heart Association/American Stroke Association encouraging utilization of telehealth. This resulted in a huge shift in stroke care especially in the rural areas (19, 20). In a 3-year study of teleneurology service for community hospitals, more than 85% of consults in different subspecialties including stroke, epilepsy, and headache were managed without the need for transfer to a higher center. This also resulted in a significant cost reduction, besides being associated with more than 90% satisfaction rate among physicians (21). As a result, various subspecialties have incorporated teleneurology into their clinical practice. The International League Against Epilepsy issued recommendations and guidelines for using telehealth in epilepsy to cover the treatment gap, especially for follow-up visits (22). The same positive experience was seen in ambulatory neurosurgical care among both patients and physicians during the COVID-19 pandemic (12). In the field of pediatric neurology, there is a scarcity of research investigating physician satisfaction. Their observation is consistent with findings in other medical specialties, which also demonstrate high rates of satisfaction. However, the majority of existing studies primarily concentrate on assessing the quality of care provided and the satisfaction of caregivers, rather than examining the perceptions of physicians. It is noteworthy that caregivers exhibited a preference for in-person visits over teleconsultations. This preference aligns with the preference expressed by our physicians for in-person visits over teleconsultations (23–25).

Our study found a high level of overall satisfaction with telehealth among the participants (mean score: 25.9 out of 33 points). However, the physicians still expressed a preference for face-to-face interviews. Furthermore, only approximately one-third of the physicians reported being able to meet the needs of their patients adequately. Despite this, the physicians have expressed that telehealth facilitates the establishment of a stronger patient‒provider relationship, enables increased productivity, and seamlessly integrates into their daily workflow.

This overall high level of satisfaction aligns with prior studies that report high satisfaction rates among healthcare providers when utilizing telemedicine (16, 26–29). This finding was also demonstrated in the field of neurology across different specialties. Although the satisfaction was different among different subspecialties based on their ability to physically assess their patients, they all preferred to integrate telehealth in their future practice. The preference of in-person visits was seen in prior studies for multiple reasons, mostly due to the difficulty in examining the patient physically (12, 30). It is noteworthy that patients and caregivers exhibited the same preference for in-person visits in prior studies despite the high level of satisfaction with teleconsultations (23, 30, 31). Given the requirement of a comprehensive physical examination for ensuring the thoroughness of the interview, this is particularly relevant in the field of neurology, especially during the initial encounters. Moreover, certain subspecialties, such as spine surgery and neuromuscular neurology, require a greater emphasis on physical assessments compared with others, such as headache specialization. Tropea et al. observed that providers specializing in headache, neuroimmunology, and movement disorders reported the highest levels of positive experiences, whereas those specializing in neuromuscular disorders reported the lowest levels (32). Kirby et al. in sport medicine further indicated that physicians felt that physical examinations conducted through telemedicine were not fully effective and that they were not reasonably confident in their consultations and diagnoses (29). Based on these findings, telehealth in neurology may demonstrate greater suitability for follow-up patient visits, as opposed to initial encounters that require a comprehensive physical assessment. Furthermore, the integration of teleneurology may be comparatively smoother in certain subspecialties when compared with others (12, 22, 32).

Furthermore, working in an academic center or private hospital was associated with a higher satisfaction rate. While approximately 70% of our sample worked at governmental hospitals, it is very difficult to conclude on this finding. In addition, the difference was very small (mean score: 27 vs. 26 out of 33). Generally, in our study, all subgroups showed great satisfaction with the services they provided using telehealth, since 60% of them were happy with the outcome of their work using technology, which was strikingly similar to the finding of Berevic et al. (16). Since our study was conducted during the COVID-19 pandemic when physicians did not have a choice apart from telehealth, further studies after the pandemic could better evaluate the usefulness and extent of the utilization of telehealth in neurology.

### Limitations

As expected from a study of this type, there are a few limitations. First, we acknowledge that telehealth modalities differ significantly, making comparisons difficult. Consequently, in our study, we primarily measured providers' satisfaction with the use of telehealth rather than the type of modality, as they differ between hospitals and regions of the Kingdom of Saudi Arabia. Another limitation is that providers' knowledge about using telehealth modalities varies, and our study did not focus on asking questions about this, which could influence the results.

## Conclusion

Our study found high overall satisfaction with telehealth among participants in neurology. While physicians expressed a preference for face-to-face interviews and reported challenges in meeting patient needs adequately, telehealth was seen as facilitating a stronger patient‒provider relationship, enhancing productivity, and integrating well into daily workflows. The satisfaction levels align with previous studies in neurology and other specialties, although physical examination requirements remain a consideration. Telehealth may be more suitable for follow-up visits rather than initial encounters, and its integration varies across subspecialties. Working in academic or private settings was associated with higher satisfaction rates, while the impact of working in governmental hospitals requires further investigation. Future studies should explore telehealth utilization beyond the pandemic and its implications for neurology practice.

## Data Availability

The raw data supporting the conclusions of this article will be made available by the authors, without undue reservation.

## References

[1] World Health Organization. Global Diffusion of eHealth: Making Universal Health Coverage Achievable: Report of the Third Global Survey on eHealth. Geneva: World Health Organization (2016). Available at: https://iris.who.int/handle/10665/252529

[2] Wechsler LR. Advantages and limitations of teleneurology. JAMA Neurol. (2015) 72:349–54. 10.1001/jamaneurol.2014.3844 25580942

[3] Dorsey ER Glidden AM Holloway MR Birbeck GL Schwamm LH. Teleneurology and mobile technologies: the future of neurological care. Nat Rev Neurol. (2018) 14:285–97. 10.1038/nrneurol.2018.31 29623949

[4] Levine SR Gorman M. “Telestroke”: the application of telemedicine for stroke. Stroke. (1999) 30(2):464–9. 10.1161/01.str.30.2.464 9933289

[5] Fonarow GC Smith EE Saver JL Reeves MJ Hernandez AF Peterson ED Improving door-to-needle times in acute ischemic stroke. Stroke. (2011) 42:2983–9. 10.1161/STROKEAHA.111.621342 21885841

[6] Fassbender K Grotta JC Walter S Grunwald IQ Ragoschke-Schumm A Saver JL. Mobile stroke units for prehospital thrombolysis, triage, and beyond: benefits and challenges. Lancet Neurol. (2017) 16:227–37. 10.1016/S1474-4422(17)30008-X 28229894

[7] Nelson RE Saltzman GM Skalabrin EJ Demaerschalk BM Majersik JJ. The cost-effectiveness of telestroke in the treatment of acute ischemic stroke. Neurology. (2011) 77(17):1590–8. 10.1212/WNL.0b013e318234332d 21917781 PMC3198982

[8] Mohamed A Elsherif S Legere B Fatima N Shuaib A Saqqur M. Is telestroke more effective than conventional treatment for acute ischemic stroke? A systematic review and meta-analysis of patient outcomes and thrombolysis rates. Int J Stroke. (2023) 27:17474930231206066. 10.1177/17474930231206066 PMC10903130 37752674

[9] Grossman SN Han SC Balcer LJ Kurzweil A Weinberg H Galetta SL Rapid implementation of virtual neurology in response to the COVID-19 pandemic. Neurology. (2020) 94:1077–87. 10.1212/WNL.0000000000009677 32358217

[10] Chua R Craig J Wootton R Patterson V. Randomised controlled trial of telemedicine for new neurological outpatient referrals. J Neurol Neurosurg Psychiatry. (2001) 71:63–6. 10.1136/jnnp.71.1.63 11413265 PMC1737484

[11] Rametta SC Fridinger SE Gonzalez AK Xian J Galer PD Kaufman M Analyzing 2,589 child neurology telehealth encounters necessitated by the COVID-19 pandemic. Neurology. (2020) 95(9):e1257–66. 10.1212/WNL.0000000000010010 32518152 PMC7538222

[12] Mohanty A Srinivasan VM Burkhardt J-K Johnson J Patel AJ Sheth SA Ambulatory neurosurgery in the COVID-19 era: patient and provider satisfaction with telemedicine. Neurosurg Focus. (2020) 49:E13. 10.3171/2020.9.FOCUS20596 33260126

[13] Alyoubi R Kobeisy S Elkady A Bamusa M Alotaibi S Muthaffar O Implementation of virtual consultations for epilepsy during the COVID-19 pandemic among neurologists in Saudi Arabia. Med Sci. (2020) 24:3717–23.

[14] Mubaraki AA Alrabie AD Sibyani AK Aljuaid RS Bajaber AS Mubaraki MA. Advantages and disadvantages of telemedicine during the COVID-19 pandemic era among physicians in Taif, Saudi Arabia. Saudi Med J. (2021) 42:110–5. 10.15537/smj.2021.1.25610 33399180 PMC7989314

[15] Abdel Nasser A Mohammed Alzahrani R Aziz Fellah C Muwafak Jreash D Talea A Almuwallad N Measuring the patients’ satisfaction about telemedicine used in Saudi Arabia during COVID-19 pandemic. Cureus. (2021) 13(2):e13382. 10.7759/cureus.13382 33754105 PMC7972323

[16] Becevic M Boren S Mutrux R Shah Z Banerjee S. User satisfaction with telehealth: study of patients, providers, and coordinators. Health Care Manag (Frederick). (2015) 34:337–49. 10.1097/HCM.0000000000000081 26506296

[17] Cohen-Schotanus J van der Vleuten CPM. A standard setting method with the best performing students as point of reference: practical and affordable. Med Teach. (2010) 32:154–60. 10.3109/01421590903196979 20163232

[18] Agarwal S Warburton EA. Teleneurology: is it really at a distance? J Neurol. (2011) 258:971–81. 10.1007/s00415-011-5920-5 21311906

[19] European Stroke Organisation (ESO) Executive Committee, ESO Writing Committee. Guidelines for management of ischaemic stroke and transient ischaemic attack 2008. Cerebrovasc Dis. (2008) 25(5):457–507. 10.1159/000131083 18477843

[20] Schwamm LH Holloway RG Amarenco P Audebert HJ Bakas T Chumbler NR A review of the evidence for the use of telemedicine within stroke systems of care. Stroke. (2009) 40:2616–34. 10.1161/STROKEAHA.109.192360 19423852

[21] Harper K McLeod M Brown SK Wilson G Turchan M Gittings EM Teleneurology service provided via tablet technology: 3-year outcomes and physician satisfaction. Rural Remote Health. (2019) 19:4743. 10.22605/RRH4743 30825873

[22] Samia P Sahu JK Ali A Caraballo RH Chan J Coan AC Telemedicine for individuals with epilepsy: recommendations from the International League Against Epilepsy Telemedicine Task Force. Seizure. (2023) 106:85–91. 10.1016/j.seizure.2023.02.005 36803864

[23] Golebiowska B Golebiowska M. Pediatric neurology and telehealth before and during SARS-CoV-2 pandemic. J Educ Health Sport. (2021) 11:254–72. 10.12775/JEHS.2021.11.12.019

[24] Sharawat IK Panda PK. Caregiver satisfaction and effectiveness of teleconsultation in children and adolescents with migraine during the ongoing COVID-19 pandemic. J Child Neurol. (2021) 36:296–303. 10.1177/0883073820968653 33170754

[25] Desai D Madaan P Dhir P Devi N Suthar R Saini AG Care of children with infantile epileptic spasms syndrome and applicability of telemedicine amidst the COVID-19 pandemic. Indian J Pediatr. (2023) 90:1254–6. 10.1007/s12098-023-04735-1 37450249

[26] Byrne E Watkinson S. Patient and clinician satisfaction with video consultations during the COVID-19 pandemic: an opportunity for a new way of working. J Orthod. (2021) 48:64–73. 10.1177/1465312520973677 33251951

[27] Andrews E Berghofer K Long J Prescott A Caboral-Stevens M. Satisfaction with the use of telehealth during COVID-19: an integrative review. Int J Nurs Stud Adv. (2020) 2:100008. 10.1016/j.ijnsa.2020.100008 33083791 PMC7564757

[28] Buchalter DB Moses MJ Azad A Kirby DJ Huang S Bosco JA III Patient and surgeon satisfaction with telehealth during the COVID-19 pandemic. Bull Hosp Jt Dis (2013*)* 78: 227–35. 33207143

[29] Kirby DJ Fried JW Buchalter DB Moses MJ Hurly ET Cardone DA Patient and physician satisfaction with telehealth during the COVID-19 pandemic: sports medicine perspective. Telemed E-Health. (2021) 27:1151–9. 10.1089/tmj.2020.0387 33512302

[30] Park HY Kwon YM Jun HR Jung SE Kwon SY. Satisfaction survey of patients and medical staff for telephone-based telemedicine during hospital closing due to COVID-19 transmission. Telemed E-Health. (2021) 27:724–32. 10.1089/tmj.2020.0369 PMC8290303 33216710

[31] James JG Park J Oliver A Xie SX Siderowf A Spindler M Linked patient and provider impressions of outpatient teleneurology encounters. Neurol Clin Pract. (2023) 13(3):e200159. 10.1212/CPJ.0000000000200159 37153752 PMC10155606

[32] Tropea TF Fuentes A Roberts Z Spindler M Yuan K Perrone C Provider experience with teleneurology in an academic neurology department. Telemed E-Health. (2022) 28:374–83. 10.1089/tmj.2021.0096 PMC9022168 34077285

